# On the Security and Data Integrity of Low-Cost Sensor Networks for Air Quality Monitoring

**DOI:** 10.3390/s18124451

**Published:** 2018-12-16

**Authors:** Lan Luo, Yue Zhang, Bryan Pearson, Zhen Ling, Haofei Yu, Xinwen Fu

**Affiliations:** 1 Department of Computer Science, University of Central Florida, Orlando, FL 32816, USA; lukachan@knights.ucf.edu (L.L.); bpearson@knights.ucf.edu (B.P.); xinwenfu@ucf.edu (X.F.); 2 College of Information Science and Technology, Jinan University, Guangzhou 510632, China; zyueinfosec@gmail.com; 3 School of Computer Science and Engineering, Southeast University, Nanjing 211189, China; 4 Department of Civil, Environmental and Construction Engineering, University of Central Florida, Orlando, FL 32816, USA; Haofei.Yu@ucf.edu

**Keywords:** IoT, data integrity, low-cost sensor, air quality monitoring, MITM

## Abstract

The emerging connected, low-cost, and easy-to-use air quality monitoring systems have enabled a paradigm shift in the field of air pollution monitoring. These systems are increasingly being used by local government and non-profit organizations to inform the public, and to support decision making related to air quality. However, data integrity and system security are rarely considered during the design and deployment of such monitoring systems, and such ignorance leaves tremendous room for undesired and damaging cyber intrusions. The collected measurement data, if polluted, could misinform the public and mislead policy makers. In this paper, we demonstrate such issues by using a.com, a popular low-cost air quality monitoring system that provides an affordable and continuous air quality monitoring capability to broad communities. To protect the air quality monitoring network under this investigation, we denote the company of interest as a.com. Through a series of probing, we are able to identify multiple security vulnerabilities in the system, including unencrypted message communication, incompetent authentication mechanisms, and lack of data integrity verification. By exploiting these vulnerabilities, we have the ability of “impersonating” any victim sensor in the a.com system and polluting its data using fabricated data. To the best of our knowledge, this is the first security analysis of low-cost and connected air quality monitoring systems. Our results highlight the urgent need in improving the security and data integrity design in these systems.

## 1. Introduction

As one of the largest environmental health risk factors, exposure to air pollution, particularly indoor air pollution, is associated with millions of premature deaths every year worldwide [1,2,3,4,5]. In fact, air pollution is now considered as the second leading cause of deaths due to non-communicable disease, only exceeded by tobacco smoking [6].

For proper management of air quality, the availability of measurement data on the spatial and temporal distributions of pollution concentrations is critical. Currently, concentrations of criteria pollutants are collected by regulatory agencies at a network of stationary monitoring stations using federal reference methods (FRM) or federal equivalent methods (FEM). Instruments with FRM or FEM designations have been extensively evaluated using strict testing protocols [7], and are able to produce reliable concentration measurements. However, these instruments are generally bulky, expensive, and require frequent calibrations and specialized personnel to operate. Due to the cost involved, the number of stationary monitoring stations is limited geographically, though air pollutant concentrations are known to vary drastically at fine scale [8,9].

With the rapid technology advancements, low-cost and portable air pollution monitoring systems have gained much attention in the past few years [7,10], and they are creating a paradigm shift in the field of air pollution monitoring [11]. Though low-cost air quality sensors are less accurate, precise and reliable compared with FRM and FEM instruments, their performances are still generally good enough for qualitative characterization of air quality status, and they only cost a fraction of the cost of FRM or FEM instruments [7,12,13,14,15,16]. Further, these systems are generally easy to use and require a minimum amount of maintenance.

In recent decades, the development of Internet of Things (IoT) accelerates continually. IoT is considered as indispensable to the future network and is gradually entering our daily routine. With its basic concept of connecting any device and even virtual objects to the Internet without human involvement [17,18], IoT based systems become “smart” and globally pervasive. The emerged air quality sensors are essentially IoT devices since they are connected to the Internet, and may be remotely configured and controlled. These sensors can be quickly deployed to establish a connected air quality monitoring network to characterize spatio-temporally resolved pollutant concentration variations at local scale, and provide opportunities to considerably enhance the capabilities of existing monitoring networks [7,10,11,19]. Therefore, these systems are increasingly being used by local government and non-profit organizations to inform the public, and to support decision makings related to air quality [11,20,21].

In regulatory monitoring activities, cyber security is an important consideration. For example, most regulatory monitoring sites are isolated from public access, and the entire process of data collection, such as data transmission and storage, are usually handled via secured protocols and networks. Security is also an important consideration for research focusing on connected IoT devices [22] in the field of computer science. Much work has been done on security enhancement of IoT devices, such as encryption algorithms, communication protocols, front-end sensor data protection, and back-end IT system protection [17,18,23,24,25,26,27] while there is no consensus on a generic framework securing IoT systems given the varieties and scales of IoT systems, and much research on IoT security and privacy is desired. For example, we have exploited various vulnerabilities in smart plugs and cameras [28,29].

We find that data integrity and system security are rarely considered during the design and deployment of low-cost and connected air quality monitoring systems, and such ignorance leaves tremendous room for unwelcome and damaging cyber intrusions. If the collected measurement data is polluted by intruders, it could misinform the public, mislead policy makers, and result in undesired outcomes.

In this study, we use the a.com system to demonstrate the potentially severe consequences of cyber security issues in low-cost sensor networks for air quality monitoring. Among many available low-cost and connected air quality monitoring platforms [30,31,32,33], a.com is one of the most popular, and one of the largest systems that provide affordable and continuous air quality monitoring capabilities to broad communities. The a.com system is able to measure ambient mass and particle number concentrations of PM2.5 (particulate matter with the aerodynamic diameter less than 2.5 μm), a critical air pollutant regulated under the National Ambient Air Quality Standards. PM2.5 is considered to be one of the most harmful air pollutants and contributes to millions of premature deaths annually worldwide [2,34]. Concentrations of PM1 and PM10 are also measured by a.com, and are processed the same way as PM2.5 in this system. Therefore, any research results based on PM2.5 are applicable to other measurements. Without loss of generality, we restrict our analysis on PM2.5 for simplification while the analysis and results are also valid for other measurements as well as PM2.5.

The major contributions of this paper can be summarized as follows. We present a systematic analysis of cyber security and data integrity of the a.com system. We first explore the the system architecture and its communication protocols based on traffic analysis using mitmproxy [35], which is a HTTPS proxy tool. We find that the system adopts unencrypted communications and uses MAC addresses to identify sensors in the sensor data sent to the web servers. This practice allows us to “pollute” sensor data by conducting a man-in-the-middle (MITM) attack or by sending fabricated data along with a victim sensor’s MAC address to the web servers. We also notice that the web servers allow us to check if a specific MAC address exists in the system. This enables us to, technically, enumerate all valid MAC addresses of a.com sensors and potentially pollute data from every sensor deployed globally. We perform extensive empirical experiments to demonstrate the feasibility of this attack. Guidelines of securing an air quality monitoring system are also discussed. To the best of our knowledge, this is the first security analysis of an IoT based air quality monitoring network. It’s also worth mentioning that we perform all the analysis only on sensors we purchased directly from a.com; thus, there are no legal issues for such experiments. We have notified a.com about the vulnerabilities of their system and are working with a.com to patch the system.

The rest of this paper is organized as follows. In Section 2, we present our materials and methods including the methodology of exploring the a.com system, the discovered system architecture as well as its communication protocol, and the methodology of polluting a.com sensor data. We evaluate the pollution results in Section 3. To defeat the polluting attack, we discuss defense measures in Section 4. The Section 5 concludes the paper.

## 2. Materials and Methods

In this section, we first present an overview of the a.com system from a user’s perspective, and introduce our methodology of exploring the system. We then present the discovered architecture of the a.com system and its communication protocol. Following that, we introduce the a.com sensor data pollution methodology.

### 2.1. Overview of the a.com System

Before introducing the exploration methodology and the discovered a.com system architecture, we first provide an overview of the a.com system including the sensor, sensor setup process, and the system view from an end user’s perspective.

#### 2.1.1. A-II Dual Laser Sensor

At the core of the a.com system is the a.com low-cost sensor. A a.com sensor is able to continuously monitor multiple environmental and air quality related metrics, including temperature, humidity and PM2.5. On the a.com website, the vendor provides three different types of sensors for choice. In this paper, we perform our analysis using the A-II model sensor, which uses two laser particle counters to provide two independent parallel channels (named channel A and channel B in this paper) of real time measurements and is the recommended sensor model from a.com. To protect the air quality monitoring network under this investigation, we denote the sensor model as A-II.

#### 2.1.2. Sensor Setup

To enable the sensor for the whole monitoring system, a user shall follow the initialization procedure as described below.

1. **Installation**: A sensor can be installed either inside or outside. To keep a sensor working in good condition and producing precise sensor outputs, the user should follow instructions provided by a.com for finding a suitable installation location. Also, a sensor shall be able to connect to a stable WiFi connection if Internet access is desired.
2. **WiFi Configuration**: Once powered up, the sensor starts to act as an access point (AP) named “AirMonitor_xxx”, where “xxx” is specific to each sensor. Users can connect to it via computer or smartphone and access the sensor’s web user interface (UI). On the web UI, users are able to provide home WiFi configuration information, after receiving which, the sensor will stop its AP mode and connect to the WiFi for Internet access. The home WiFi configuration is saved in the sensor for future automatic connections.
3. **Sensor Registration**: To conveniently check a sensor’s collected data and utilize the graphical presentation of historical sensor data provided by the a.com Map web application, a user is required to conduct a prior online sensor registration at the a.com website, submitting important sensor information including sensor name, sensor geographical location, sensor MAC address, and a specific user’s email. The provided email must match the one used during sensor purchase, otherwise the registration would be denied. The requirement of providing the associated user email is a prevention measure for avoiding unauthenticated sensor registration and utilization by malicious attackers even if they somehow possess the sensor MAC address. After completing the registration process, sensor data will be visualized on the a.com Map at the registered geographical location.

#### 2.1.3. User-Perspective Architecture

**Figure 1:**
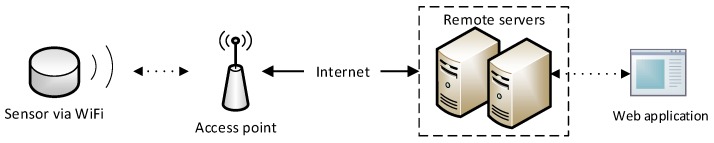
System Architecture from User’s Perspective.

After successfully setting up the sensor, by observing the a.com system’s operating mechanism, we can obtain a preliminary view of the system architecture from a user’s perspective. We illustrate this architecture in Figure 1.

From a user’s perspective, a.com can be recognized as an IoT system that consists of user-end air quality monitoring sensors and remote servers. The sensor connects to the user provided WiFi router, via which it then accesses the Internet. Utilizing the network connection, the sensor automatically uploads air quality measurement data to the remote servers.

For viewing uploaded sensor data, a.com provides users with the following options.

1. **From a.com Map:** In this option, users are able to use a web application called “Map”, which is an integrated map system overlaid with all public sensors located at their reported geographical locations. Users can use the sensor geographical location or sensor name to pinpoint the desired sensor unit. In the “Map” application, a user is able to view both real-time and historical statistical sensor data in numerical and graphical presentations.
2. **From downloaded csv files**: In the second option, there exists a webpage for cvs format historical sensor data downloading. For each sensor unit, data items appear at an interval of approximately 80 s and are divided into four files, with two for each sensor channel.
3. **From sensor’s web UI**: The last option for viewing sensor data is accomplished via a sensor’s web UI. When a user connects to the sensor directly at bootstrapping (before connecting it to the Internet), a user can also access a web interface that shows real time sensor measurements. Nonetheless, this approach has its deficiencies: First, the sensor is disconnected from the Internet and the a.com servers, interrupting data uploading and resulting in a permanent gap of the server’s historical sensor data collection. Second, this option does not have any historical or graphical data illustration accessibility.

**Figure 2:**
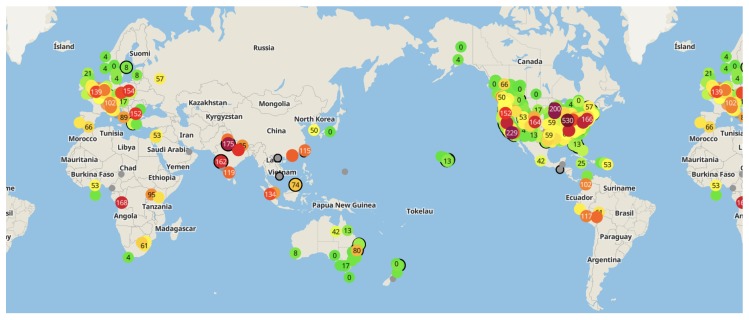
Screenshot from the a.com Map in reference for application popularity.

We believe that most users shall adopt the a.com Map application as it is the most convenient and intuitive sensor data inspection choice. This observation is further consolidated by the popularity of publicly visible sensors on the a.com Map application, as presented in Figure 2.

A system view from the user perspective is precious in capturing a foundation of the system architecture. Nonetheless, the lack of sufficient details and insights obstructs a comprehensive understanding of the system and the exposure of its security vulnerabilities. It is evidently worth paying more efforts in digging into a more complete and detailed system architecture, deeper than the plain perspective view from daily usage. We therefore present our exploration methodology in the following section.

### 2.2. Methodology of Exploring a.com System

**Figure 3:**
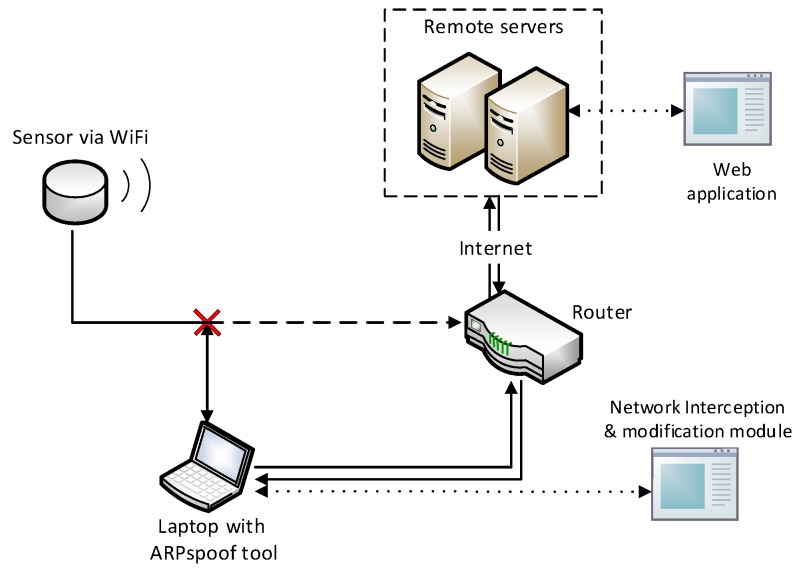
Experiment Setup.

To explore the architecture of the a.com system, we construct an experimental environment capable of capturing network traffic between a.com sensors and their servers, as shown in Figure 3. Firstly, we establish a wireless local area network (WLAN) using a wireless router with Internet access. A sensor is configured to connect to this router wirelessly. Secondly, a laptop installed with an ARP spoofing tool, the “ARPspoof” from the “dsniff” tool package [36], is connected to the same WLAN. ARP spoofing is an attack methodology where Address Resolution Protocol (ARP) messages with false association of a target IP address to the attacker’s MAC address are fabricated and broadcasted, with the purpose of redirecting network traffic of interest from the original destination to the attacker, such that communication interception and alteration could be performed. The ARPspoof tool is configured to reroute all communications between the a.com sensor and its web servers to the laptop for network traffic analysis, interception, and possible modification.

Through this environment setup, we can successfully modify the WLAN topology without any change of physical network connections for enabling the functionality of the network interception and modification module. This enables the possibility of capturing sensor network traffic, inspecting the sensor message structure, and carrying out MITM attack against the sensor. Messages originated from and destined to the sensor can now be freely monitored and modified at the laptop if necessary. Additionally, to assist with the initial message inspection, we install Wireshark [37], a network protocol analyzer, on the laptop to capture and analyze network traffic of the sensor for further exploration.

By analyzing the captured traffic packets, we find that the a.com sensors use HTTP as the communication protocol. This drives us to choose mitmproxy [35] for further network traffic analysis. Mitmproxy is a HTTP/HTTPS proxy that can perform the MITM attack, where attackers hijack an ongoing network communication and perform network traffic analysis or information alteration. With its Python scripting API, a user is able to execute customized scripts and manipulate network traffic automatically. Here, we run mitmproxy in its transparent mode, which does not require particular configuration of the sensor of interest. We are able to observe corresponding HTTP requests and responses, and changes reflected on the map for modified messages.

### 2.3. Discovered System Architecture

**Figure 4:**
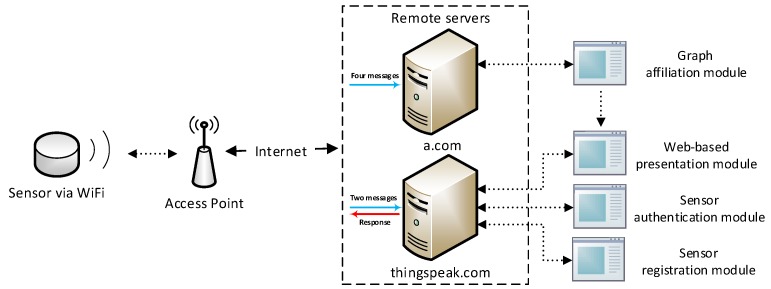
Discovered System Architecture.

Through interception, analysis, and experimental modification of network communications, we are able to identify three key components of the a.com system architecture shown in Figure 4: sensors (described previously), servers, and a map.

**Servers:** The a.com system utilizes two servers: www.a.com and api.thingspeak.com (a data analytics platform). The first server is used to verify sensor identity and other related parameters, perform numerical calculations of sensor data, and respond to sensor requests with informative messages. The second server, however, is mostly used for data analysis and visualization purposes.

**Map:** The a.com Map is a web-based map application built on Google Maps. It allows end users to access real-time and historical data from connected sensors, in both numerical and graphical illustrations. According to our analysis, these data are generated independently from corresponding servers: the numerical data is originated from www.a.com, while the graphical data presented in a line chart is supplemented by api.thingspeak.com and renewed approximately every 10 min. Data from each sensor is automatically matched to the sensor’s self-reported geographical location during registration.

#### 2.3.1. Discovered Communication Protocol

**Figure 5:**
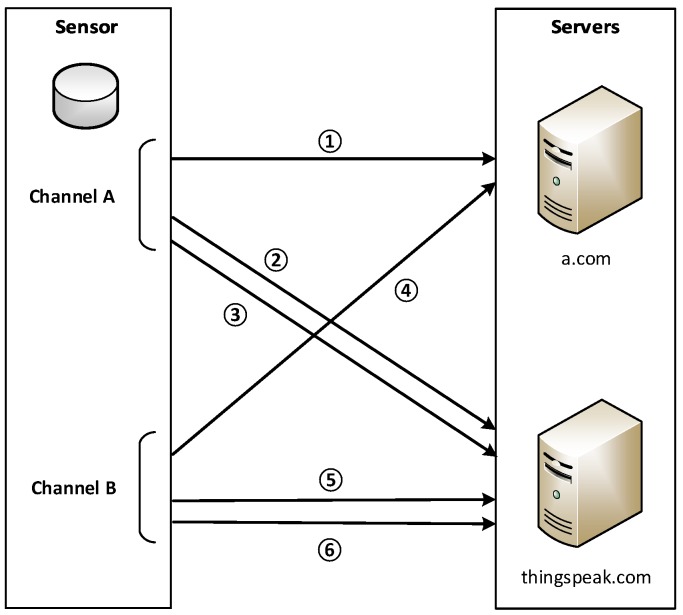
Communication Protocol between an a.com Sensor and Servers.

After analyzing the captured traffic between the A-II sensor and its servers, we also discover a few characteristics about sensor communication: Firstly, the communications between a sensor and servers are not encrypted. Messages sent by a sensor use the HTTP GET request method. Secondly, a sensor communicates with its servers automatically in a periodical pattern. In each period, a A-II sensor constructs six HTTP non-persistent connections with three for each independent sensor channel (corresponding to each independent particle counter inside the sensor) as shown in Figure 5. Messages constructed for each channel are identical in structure. Thirdly, for three messages from a channel in each communication period: the first message contains measurement data of PM2.5 and two API keys, and is sent to www.a.com with the sensor’s MAC address as identification; the other two messages contain measurement data and API keys, and are sent to api.thingspeak.com. ThingSpeak further allocates two channels for each independent particle counter in the sensor (thus four ThingSpeak channels in total for a sensor unit with dual laser particle counters). To upload data to the ThingSpeak server, each ThingSpeak channel will be bonded with a unique API key, which is used for identity verification. Therefore, only with a correct key contained in the message can a sensor upload data through the corresponding ThingSpeak channel.

#### 2.3.2. Message Composition

To better understand the message composition, the structure of these messages is now stated as follows. Without loss of generality, we use three messages for channel A as an example, and provide message notations for later reference:

- Message **M-1A**: A sensor constructs a HTTP connection to www.a.com and sends a HTTP GET request which contains the sensor MAC address, two keys for sensor channel A (named K-1A and K-2A) used in later communication with api.thingspeak.com, and all measurement data from sensor channel A. The MAC address is used for one-way device identification and the server will respond with a message corresponding to the validity of the MAC and the ThingSpeak keys.
- Message **M-2A**: A sensor connects to api.thingspeak.com and sends a request with K-1A and a part of measurement data from channel A for data analysis and visualization purposes.
- Message **M-3A**: A sensor connects to api.thingspeak.com again, but sends a HTTP request with K-2A and the remaining measurement data from channel A not involved in M-2A.

The same pattern will be utilized again for constructing three non-persistent HTTP connections (M-1B to M-3B) for channel B with corresponding ThingSpeak keys K-1B and K-2B. Each communication period of six messages lasts about 80 s, with three channel A messages coming first, followed by three channel B messages.

#### 2.3.3. Data Format and AQI

Comprehensive understanding of data formats embedded in communication messages between a sensor and servers is essential for both system architecture analysis and sensor data pollution. Recall that the HTTP GET request is used by sensors. In such a request, sensor data is presented in the header section with a “name: value” pair format. These headers are stored in the URL of the HTTP request, hence can be easily identified.

As aforementioned, three respective messages for channels A and B are entirely identical in structure. Therefore, when dissecting internal data formats of the messages for each communication period, it suffices to look at the three unique data formats derived from three messages of each channel. We depict these three unique data formats in Table 1 for messages M-1A and M-1B, Table 2 for messages M-2A and M-2B, and finally Table 3 for messages M-3A and M-3B.

From the internal data format as presented in Table 1, Table 2 and Table 3, we are capable of obtaining a well-rounded observation of data communication between sensors and servers. We also discover that sensor data presentation on the a.com Map not only uses raw sensor data, but also analytic results calculated from raw sensor data. In fact, Air Quality Index (AQI), a major quantitative index for determining air quality, is not transferred via direct communication between a sensor and servers. The a.com claims to use the “Federal Environmental Protection Agency (EPA) Air Quality Index (AQI) scale”. Accordingly, we present the AQI calculation formula in Equation (1) [38]:

(1) $$AQI=\frac{I_{high}-I_{low}}{C_{high}-C_{low}}\left(C-C_{low}\right)+I_{low},$$

where *C* is the pollutant concentration, $C_{low}$ is the concentration breakpoint that is ≤C, $C_{high}$ is the concentration breakpoint that is ≥C, $I_{low}$ is the index breakpoint corresponding to $C_{low}$, and $I_{high}$ is the index breakpoint corresponding to $C_{high}$.

Furthermore, as expressed in Equation (1), AQI is computed based on a selected reference pollutant. We discovered that a.com calculates two separate AQI values using PM2.5 and PM10 by the same formula (with corresponding parameters). The data pollution mechanisms for PM2.5 and PM10 are identical. Therefore, we restrict our later description and experiments to PM2.5.

#### 2.3.4. Server Response Format

The www.a.com server would send back HTTP responses when they receive HTTP requests originated from the a.com sensor. Exemplar message exchanges are presented in Table 4. If a message is sent to the server with both correct sensor MAC address and correct keys, the server will send back the geographic coordinates of this registered sensor. If the MAC address is correct but the keys are invalid, the response will return both the geographic coordinates and the correct keys for future communication between the sensor and the api.thingspeak.com server. Finally, If the MAC address is invalid, the server responds with “NOT FOUND”, indicating that this MAC address does not belong to any registered a.com sensor. These responses enable us to verify possible sensor MAC addresses and also obtain their corresponding ThingSpeak keys.

### 2.4. Methodology on Polluting the a.com System

In this section, we introduce data pollution methodology against the a.com system in different scenarios. We first introduce how to pollute data of a victim sensor if we physically possess the sensor, or if the victim sensor’s MAC address is known. We then discuss how to enumerate the MAC addresses of every a.com sensor so that we can pollute any sensor of the a.com system.

#### 2.4.1. Possessing a Sensor

As a rudimentary case, the first scenario (denoted as Scenario A) for data pollution is that we physically possess the a.com sensor. Recall in Section 2.3.1, we identify the following features which contribute to data pollution: Firstly, the network traffic between a.com sensors and servers are in plaintext; secondly, only the sensor MAC address is used for identification. Therefore, it suffices to use the same environment setup as in Figure 3, which is used in system architecture discovery as well, for launching the pollution.

According to the experiment setup, the data pollution can be sufficiently accomplished by the MITM attack. Through the environment setup, we are capable of intercepting messages between a sensor and its servers and modifying them freely as needed. To accomplish this in practice, the HTTP proxy tools such as mitmproxy (on Linux) and Fildder (on Windows) can be used. Such tools are employed for listening to bidirectional sensor-server communications, and plug-in scripts are used to manipulate messages automatically in these communications. If the message contains raw sensor data of interest, we modify the message with selected data values before forwarding it to the original server destination. Since sensor data is stored in the HTTP request header when uploaded in the clear data format as mentioned in Section 2.3.3, locating specific sensor data by corresponding header name and rewriting it to any value we desire is a sound approach with no apparent obstacles.

#### 2.4.2. Knowing a MAC

In this scenario (denoted as Scenario B), physical access to an a.com sensor is no longer permitted. A plausible approach is to fabricate all messages from scratch to imitate the victim sensor if we know the MAC address of the victim sensor.

There are indeed multiple ways to obtain a sensor’s MAC address without physical possession. We now present one representative approach with only publicly available information within the scope of the a.com system. In this approach, we utilize the observable geographical locations of registered sensors on the a.com Map. Thus the issue becomes, given the geographical location of a sensor, without direct physical access to the sensor and the possibility of direct network manipulation, can its MAC address still be attained? To carry out this task, we leverage wardriving, which refers to scanning and sniffing for WiFi network information in a moving vehicle by using a laptop or other computer devices.

In demonstration, we ask a volunteer to set up an a.com sensor within his/her household. We know no information for the sensor of interest, including its MAC address, but only the geographical location of the volunteer’s household. By wardriving around the volunteer’s household using the popular sniffing tool kismet [39], we successfully intercept WiFi network communication information in the surrounding area. Although traffic from multiple active WiFi networks are merged together through this process, the desired sensor MAC address can still be spotted with ease. This is because, by convention, the first 6 hex digits (prefixes) of the 12 digits MAC address represents a specific manufacture. The a.com sensors contain a specific WiFi microchip ESP8266 in which the prefix of the MAC address belongs to one of several pre-assigned prefixes owned by the manufacturer Espressif Systems. MAC address prefixes allocated to a vendor such as Espressif can be looked up at various websites [40], by which 24 MAC address prefixes are found given to Espressif Systems (Table 5). By using prefix patterns, we match and find the actual sensor MAC via wardriving without encountering any ambiguous situations in sensor MAC recognition during wardriving. Nonetheless, even if ambiguity appears, we can leverage message responses from the a.com server to easily distinguish the actual sensor MAC from other candidates.

Once knowing a specific registered sensor’s MAC address, we pollute its data sent to the a.com system by creating a fake sensor (a computer program), fabricate messages according to the discovered data formats, and send the fabricated data to corresponding servers. The fabricated messages will contain the victim sensor’s MAC address, and will be accepted by the servers as authentic data from the specific victim sensor. We call this attack as a spoofing attack since the fake sensor pretends to be the victim sensor and sends fake data to a.com web servers.

In the spoofing attack, a.com servers receive two sets of data for one sensor: authentic data from the victim sensor, and fabricated data from the fake sensor. The servers merge the two sets of data and use the merged data to indicate air quality. Inevitably, the authentic data from the victim sensor are “polluted” by the fake data.

**Figure 6:**
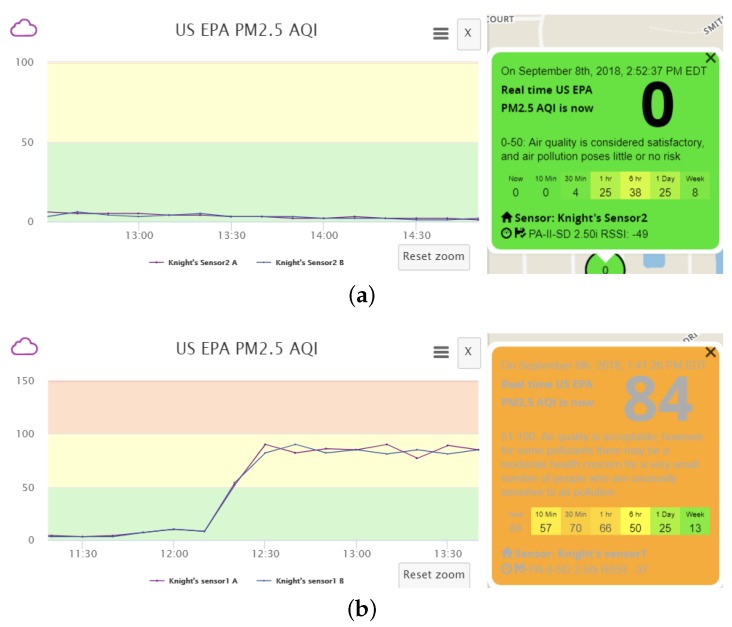
AQI fluctuation for Scenario B. (**a**) Before data pollution; (**b**) After data pollution.

In addition, we can vary the data transmission frequency of counterfeit sensor data to better suppress authentic data, to conceal malicious activities from the owners of the victim sensor and the servers, and to achieve a desired level of air pollution. We find that each of the two channels within a real sensor transmits data at an interval of approximately 80 s. If our fake sensor also sends counterfeit data every 80 s, the a.com servers would receive both authentic and fabricated sensor data at the same frequency, and the two data sets will be averaged. The resulting effect of data pollution, taking the AQI calculated based on measured PM2.5 as an example, is shown in Figure 6. One can observe two phenomena: Firstly, the polluted data presents many ups and downs. This fluctuation is the result of averaging the received real and fake measurements within each 10-min period. When the fake data sending frequency is relatively low, it is likely that the number of received fake data samples varies slightly between different periods. Meanwhile, the fake data is usually much larger (or smaller) than the real data. Hence, receiving even one more fake message may lead to apparent fluctuation in the averaged result. For the real measurements, slight fluctuation exists as well due to natural variations of pollutant concentrations in the ambient environment. Therefore, the polluted data fluctuation is actually the combined result of the variations in real data and the instability caused by received fake data. Secondly, the AQI is not the intended value, which should be 151, as suggested by the fabricated message, due to average of both authentic and fabricated data. Such phenomena, especially the data fluctuation, could raise the possibility of detection by the sensor owner or the a.com system. It is of great interest to better understand the phenomena and whether optimization procedures can be performed to reduce data fluctuation, to better suppress authentic sensor data, and to achieve a desired level of AQI.

After significant efforts in analyzing the phenomena, we arrive at the following explanation: the graphical representation of the AQI value on the a.com Map is calculated using averaged PM2.5 measurement data in the past 10 min. Numerical presentation of the AQI value follows the same computation methodology but with a varied interval.

Therefore, an attacker may want to adjust the data update frequency of the fake sensor so that the AQI can be manipulated to the desired value. Here, we define the following notations: (1) the update interval of the visualized data on map as $I_{VU}$, where $I_{VU}=10$ min =600 s; (2) the real sensor and fake sensor data update intervals as $I_{r}$ and $I_{f}$ respectively, where $I_{r}=80$ s and $I_{f}$ is to be determined; (3) the real PM2.5 measurement (assuming constant during pollution) and fabricated PM2.5 measurement as $P_{r}$ and $P_{f}$ respectively; (4) with respect to the targeted AQI, the corresponding range of PM2.5 pollutant measurement as $R_{\mathrm{PM}2.5}=\left[LB,UB\right]$ (obtainable via the United States EPA AQI calculation table [38]). Here, only $P_{f}$ and $I_{f}$ are controllable variables, and all others are given. Therefore, we define three problems for pollution methodology optimization as follows and also present the sketchy solution methodologies for these three problems.

**Problem I: Under what values of $P_{f}$ and $I_{f}$ can the visualized AQI be the same as the targeted AQI?**

**Solution.**  To ensure the displayed AQI value is the same as desired, a necessary and sufficient condition is to select proper $P_{f}$ and $I_{f}$ ($0<I_{f}\leq I_{VU}$) such that the average of all received PM2.5 measurements during each update interval is located within the range $R_{\mathrm{PM}2.5}=\left[LB,UB\right]$. This can be mathematically formulated as the following inequality.

(2) $$LB\leq \frac{P_{f}\cdot \frac{I_{VU}}{I_{f}}+P_{r}\cdot \frac{I_{VU}}{I_{r}}}{\frac{I_{VU}}{I_{f}}+\frac{I_{VU}}{I_{r}}}\leq UB.$$

 ■

Since smaller values of $I_{f}$ are equivalent to higher message transmitting frequencies, the adversary may want to know a minimum required number of messages, which corresponds to the maximum value of $I_{f}$, for a given $P_{f}$, to ensure the targeted AQI can be achieved. This can be formulated as Problem II.

**Problem II: For a given $P_{f}$, what is the maximum applicable value of $I_{f}$?**

**Solution.**  This can be viewed as an optimization problem:

$$\mathrm{Maximize}\;\;I_{f}$$

Subject to:

(3) $$\begin{array}{c} \frac{P_{f}\cdot \frac{I_{VU}}{I_{f}}+P_{r}\cdot \frac{I_{VU}}{I_{r}}}{\frac{I_{VU}}{I_{f}}+\frac{I_{VU}}{I_{r}}}\leq UB,\\ \frac{P_{f}\cdot \frac{I_{VU}}{I_{f}}+P_{r}\cdot \frac{I_{VU}}{I_{r}}}{\frac{I_{VU}}{I_{f}}+\frac{I_{VU}}{I_{r}}}\geq LB,\\ I_{f}>0,\\ I_{f}\leq I_{VU},\\ P_{r}\notin \left[LB,UB\right]. \end{array}$$

Therefore, we can derive the maximum value of $I_{f}$ (denoted as $I_{max}$) for different cases as presented by Equation (4):

(4) $$I_{max}=\left\{\begin{array}{cc} \min\left\{\frac{UB-P_{f}}{P_{r}-UB}I_{r},I_{VU}\right\}, & \;\mathrm{if}\;P_{r}>UB,\\ \min\left\{\frac{P_{f}-LB}{LB-P_{r}}I_{r},I_{VU}\right\}, & \;\mathrm{if}\;P_{r}<LB. \end{array}\right.$$

 ■

Deductions here are assuming a suitable value of $P_{f}$. Certainly, requirements exist when selecting value of $P_{f}$ to make data pollution action practical. In this work, when $P_{r}>UB$, we opt to choose $P_{f}\in \left[LB,UB\right)$; and when $P_{r}<LB$, we opt to choose $P_{f}\in \left(LB,UB\right]$. This conservative yet clear choice satisfies all requirements of $P_{f}$, and more importantly, it additionally grants that we can send fabricated messages as frequently as we want (i.e., $I_{f}$ can be selected for as close to 0 as we want, so long as it is less than $I_{max}$, targeted AQI still can be achieved). Such freedom enables the choice of any adequately desired higher fake data frequency, which can thus help to alleviate fake data receiving instability and better suppress real data and its variations, so that a better pollution result with much less fluctuation can be achieved. The case when $\;LB\leq P_{r}\leq UB$ is trivial since no fabricated message is needed for pollution in this situation.

Furthermore, we empirically consider the possibility of deciding a uniform $I_{max}$ for parallel multiple sensor data pollution, in which identical targeted AQI and a proper $P_{f}$ are used for polluting all victim sensors in parallel. This is expressed in Problem III.

**Problem III: What is the uniform $I_{max}$ when multiple sensors are being polluted simultaneously?**

**Solution.**  Denote this uniform maximum fake sensor data update interval as $I_{uni}$. We assume the number of sensors being polluted simultaneously is *n*. The corresponding real PM2.5 measurements for these sensors are $P_{r_{1}}$, $P_{r_{2}}$, …, $P_{r_{n}}$. Without loss of generality, we assume these *n* values are all in an increasing order, and among these *n* values, the first $N_{1}$ values $P_{r_{1}}$, …, $P_{r_{N_{1}}}$ are less than LB; values $P_{r_{N_{1}+1}}$, …, $P_{r_{N_{2}}}$ are in between LB and UB; and remaining values $P_{r_{N_{2}+1}}$, …, $P_{r_{n}}$ are greater than UB, where $1\leq N_{1}\leq N_{2}\leq n$. This assumption is general and can always be achieved by reordering all sensors.

Since sensors in position $N_{1}+1$ through $N_{2}$ do not have any requirement on $I_{uni}$, we exclude them from the later portion of the solution. For the rest of the sensors, we denote corresponding $I_{max}$ for sensor *i* as $I_{max_{i}}$, $S_{1}=\left\{1,\cdots ,N_{1}\right\}$, and $S_{2}=\left\{N_{2}+1,\cdots ,n\right\}$. $I_{uni}$ can be expressed as:

(5) $$\begin{array}{cc} I_{uni} & =\min\{I_{max_{i}}\;|\;i\in \left[1,n\right]\},\\ & =\min\{I_{max_{i}}\;|\;i\in S_{1}\;\cup \;S_{2}\},\\ & =\min\{I_{uni-S_{1}},I_{uni-S_{2}}\}, \end{array}$$

where $I_{uni-S_{1}}=\min\left\{I_{max_{i}}\;|\;i\in S_{1}\right\}$ and $I_{uni-S_{2}}=\min\left\{I_{max_{i}}\;|\;i\in S_{2}\right\}$. By Equation (4),

(6) $$\begin{array}{cc} I_{uni-S_{1}} & =\min\{I_{max_{i}}\;|\;i\in S_{1}\},\\ & =\min\left\{\min\left\{\frac{P_{f}-LB}{LB-P_{r_{i}}}I_{r},I_{VU}\right\}\;|\;i\in S_{1}\right\},\\ & =\min\left\{\frac{P_{f}-LB}{LB-\min\{P_{r_{i}}|i\in S_{1}\}}I_{r},I_{VU}\right\}=\min\left\{\frac{P_{f}-LB}{LB-P_{r_{1}}}I_{r},I_{VU}\right\}. \end{array}$$

Similarly,

(7) $$\begin{array}{cc} I_{uni-S_{2}} & =\min\{I_{max_{i}}\;|\;i\in S_{2}\},\\ & =\min\left\{\min\left\{\frac{UB-P_{f}}{P_{r_{i}}-UB}I_{r},I_{VU}\right\}\;|\;i\in S_{2}\right\},\\ & =\min\left\{\frac{UB-P_{f}}{\max\{P_{r_{i}}|i\in S_{2}\}-UB}I_{r},I_{VU}\right\}=\min\left\{\frac{UB-P_{f}}{P_{r_{n}}-UB}I_{r},I_{VU}\right\}. \end{array}$$

Thus,

(8) $$I_{uni}=\min\left\{I_{uni-S_{1}},I_{uni-S_{2}}\right\}=\min\left\{\frac{P_{f}-LB}{LB-P_{r_{1}}}I_{r},\frac{UB-P_{f}}{P_{r_{n}}-UB}I_{r},I_{VU}\right\}$$

That is, when launching multiple sensor pollution attacks simultaneously, a suitable uniform $I_{max}$ exists, and is only possibly affected by the smallest and largest real PM2.5 readings among all sensors. ■

#### 2.4.3. Enumerating MAC Addresses

The challenge of the previously stated data pollution approach in Section 2.4.2 is to obtain victim sensors’ MAC addresses, which requires physical access to the geographical locations of sensors. To overcome this issue, we develop an automatic, efficient, and low-cost large-scale MAC address detection strategy. We could technically obtain MAC addresses for every a.com sensor by using this strategy.

As discussed previously, responses from a.com servers automatically differentiate correct and incorrect sensor MAC addresses (Table 4). Such responses can be exploited to screen for real MAC addresses of a.com sensors. A thorough search of the entire MAC address space is theoretically possible, but practically not feasible given the enormous amount of possible MAC addresses (in the order of $16^{12}$). However, a.com sensors use the ESP8266 chip and there are 24 MAC address prefixes assigned to its manufacturer Espressif Systems as listed in Table 5. Therefore, the entire search space for sensor MAC addresses is reduced to around $24\times 16^{6}\approx 0.4$ billion.

With such a dramatically reduced search space and with possible parallel deployment of the scanning program, a large-scale automatic sensor MAC addresses screening is feasible, which could consequently enable a large-scale sensor data pollution of the entire a.com system. Technically, we could first enumerate all possible MAC addresses of all a.com sensors, and verify the authenticity of each MAC address individually by sending messages containing each individual MAC address to the www.a.com server. If a correct MAC address is sent, the server will respond with geographic coordinates of the corresponding sensor. Using this approach, we could populate a list of every a.com sensor and perform a large-scale data pollution attack on the a.com system.

Algorithm 1 presents our sensor MAC address scanning algorithm. More experiment details are included in the next section.
**Algorithm 1.** MAC scanning algorithm.
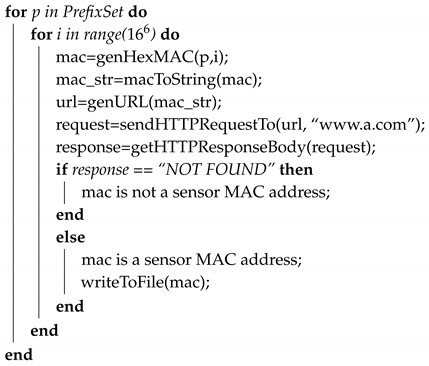


## 3. Results

In this section, we present the experiment results. These experiments show the effectiveness of our data pollution approaches, including the data pollution attacks for each of the first two scenarios, the effectiveness of the wardriving attack, and a feasibility analysis of the large-scale sensor scanning attack.

### 3.1. Effectiveness of Pollution Attack in Scenarios A & B

Either Scenario A or Scenario B focuses on the data pollution of a single target sensor. In this section, we present results for both scenarios.

#### 3.1.1. Effectiveness of Pollution Attack in Scenario A

In scenario A, we utilize mitmproxy to conduct the MITM attack between an a.com sensor and its servers. When launching the attack, all messages from the target sensor are intercepted and modified if necessary, then are redirected to their original destinations. That is, in this experiment, we only modify data sent from a real sensor but without changing its communication pattern or frequency.

**Figure 7:**
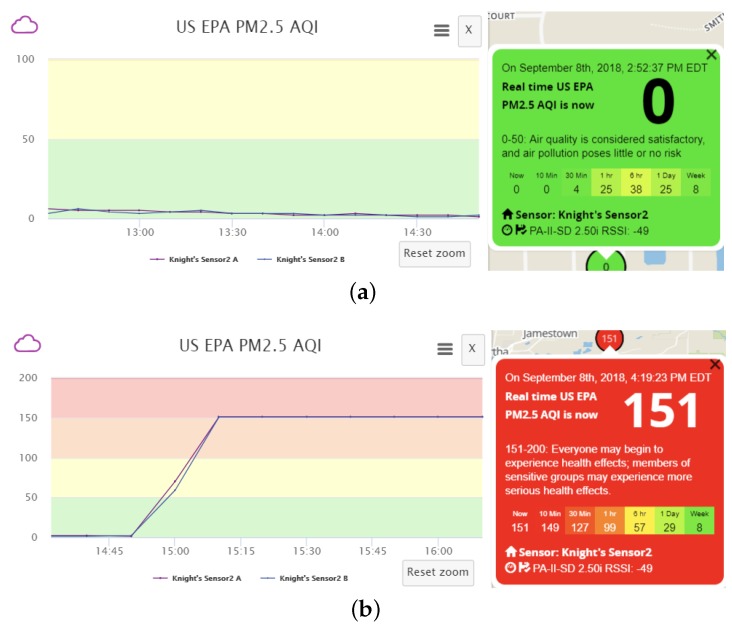
AQI fluctuation for Scenario A. (**a**) Before data pollution; (**b**) After data pollution.

As an example, the data pollution effects for increasing PM2.5 reading and the respected AQI change can be seen in Figure 7. During the experiment, ${\mathrm{AQI}}_{\mathrm{PM}2.5}$ of the real sensor remains at around 0, while our intended value of ${\mathrm{AQI}}_{\mathrm{PM}2.5}$, modified by altering the PM2.5 measurement contained in the uploaded sensor data, is 151. It can be verified that the effectiveness of data pollution is apparent, as reflected in the dramatic change of AQI. We can also verify that the data pollution result is stable and experiences no fluctuation due to the complete blocking of original sensor data. Once the polluted data can be manipulated in a stable manner, we can freely control the data fluctuations so that the shape of the curve could appear more natural and less suspicious, to further camouflage the polluted data. In this way, both the sensor owner and the a.com system have extreme difficulty in noticing the data pollution behavior and suffer from permanent loss of original sensor data.

#### 3.1.2. Effectiveness of Pollution Attack in Scenario B

During the data pollution experiment for Scenario B, ${\mathrm{AQI}}_{\mathrm{PM}2.5}$ of the real sensor remains at around 0, whereas our modified value of ${\mathrm{AQI}}_{\mathrm{PM}2.5}$, attained by conducting the spoofing attack, is 151.

**Figure 8:**
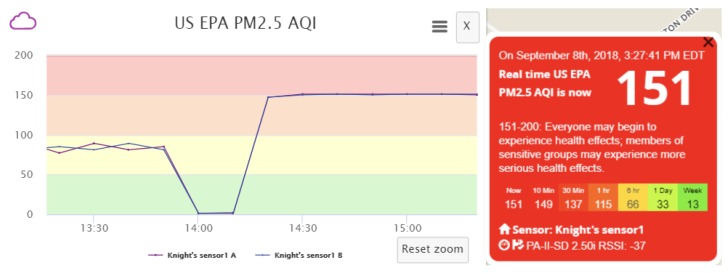
AQI fluctuation for Scenario B with optimized data pollution strategy.

As described in Section 2.4.2, the a.com servers receive two sets of data simultaneously during the spoofing attack: one authentic set of data from the target sensor, and the other fabricated data set from the fake sensor. In this case, $P_{r}$ is much smaller than LB, hence we choose $P_{f}\in \left(LB,UB\right]$, and opt to set $P_{f}=UB$. We adjust the transmission interval of the fake sensor to be slightly smaller than $I_{max}$ (computed by Equation (4)), but significantly smaller than $I_{r}$, and find that it is sufficient to suppress the authentic data and conceal the spoofing attack. The effects of this optimized polluting strategy can be observed in Figure 8. With the optimized strategy, data fluctuations are almost entirely eliminated comparing to Figure 6 and the displayed AQI result indeed reaches the intended value stably. In short, we are able to successfully attain an effective pollution methodology while the servers receive both authentic and fabricated sensor data.

### 3.2. Effectiveness of Wardriving Attack

As an auxiliary component of the data pollution approach for scenario B, the described wardriving attack is an plausible way to obtain the MAC address of a sensor by knowing only its approximate geographical location, with no requirement of other information or direct physical sensor possession.

To evaluate the effectiveness of the wardriving attack, we design the following setup: (1) All experiments are carried out by a laptop equipped with the Kismet network sniffing tool and a wireless network card. The laptop is set inside a vehicle fulfilling the wardriving technique. (2) Before the experiment, we conduct network traffic sniffing in the surrounding of the volunteer’s household several times beforehand with the activated Kismet tool for the purpose of locating an area where the targeted WiFi network can be received persistently during the experiment period. Then we start the actual experiments. (3) In each experiment, we keep the wardriving vehicle consistently within the previously identified area, activate the Kismet tool, and record capturing waiting time until the targeting sensor MAC is observed. The resulting waiting time is then recorded and the experiment starts again at another arbitrary time. (4) This independent experiment is repeated 30 times for revealing its intrinsic randomness.

**Figure 9:**
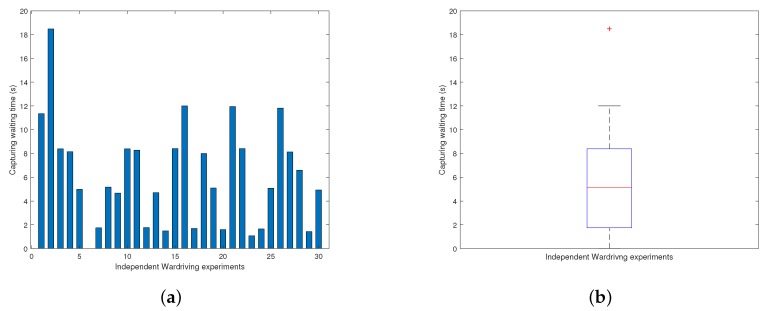
MAC capturing waiting time in wardriving experiments. (**a**) Time for each experiment; (**b**) Time distribution box plot.

The consequent experiment results, including waiting time for each experiment round and a box plot of waiting time distribution, are shown in Figure 9. It can be observed that randomness impacted the actual MAC capturing waiting time. Nonetheless, the resulting waiting time distribution remains acceptable for the wardriving attack with an average waiting time being 6.18 s, the minimum at approximately 0.006 s, and the maximum at around 18.49 s. In fact, from in-depth observation of the sensor operating mechanism, we believe the waiting time results from a combination of two factors: First, sensor messages are being sent in a predefined periodical pattern with internal temporal separations between messages; second, wardriving sniffing is only capable of capturing sensor MAC addresses when its communication occurs. Thus, it is reasonable to expect that the wardriving capturing waiting time experiences inevitable yet tolerable fluctuation.

### 3.3. Feasibility of Sensor Scanning Attack in Scenario C

We have proposed an approach for automatic large-scale sensor MAC address detection for the a.com system. Even though we are able to drastically reduce the search space by leveraging prefix patterns of sensor MAC address, the efficiency in verifying one single address is still crucial in determining the overall efficiency and feasibility of the sensor scanning attack.

**Figure 10:**
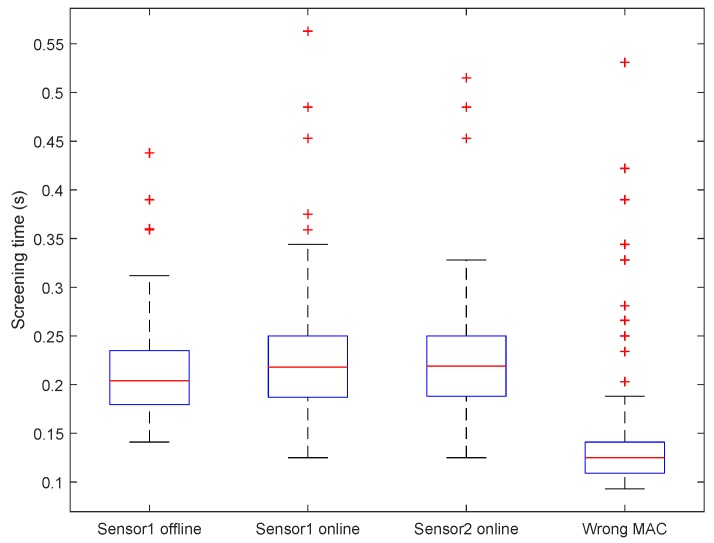
Single MAC screening time box plot figure.

In Figure 10, we present results of an empirical experiment in estimating the time cost of scanning a single MAC address. In this experiment, we utilize real sensor MAC addresses from two a.com sensors in our possession (named sensor1 and sensor2), and one invalid MAC address inside the search space altered from the MAC address of sensor1. We perform the tests under four scenarios: (1) using sensor1’s MAC when sensor1 is offline; (2) using sensor1’s MAC when sensor1 is online; (3) using sensor2’s MAC when sensor2 is online; and (4) using the invalid MAC address. For each scenario, we assemble the sensor message accordingly, send the message to the www.a.com server, and record the time it takes for the server to respond. We repeat this test 100 times for each scenario, and plot the distributions of server response time (Figure 10). It is clear that the average response time is either around or less than 0.2 s for all four scenarios, hence we choose to use the average response time of 0.2 s for later analysis.

Given its nature, the process of MAC address scanning can be easily paralleled since no data exchange nor synchronization is needed among individual parallel tasks. Here we refer to the computing unit for each parallel task as a “worker”, who is responsible for screening one MAC address at any moment. A worker can be a physical computing equipment, or virtual computing resource. With different number of available workers and assuming an average response time of 0.2 s, the time costs of scanning the entire MAC address space for a.com sensors can be estimated as in Table 6.

Table 6 reveals that with 1000 workers, the entire brute-force scanning process could be accomplished in less than one day, thus proving the feasibility of our designed large-scale sensor MAC scanning attack. Using the popular cloud computing platform Amazon EC2 [41] as an instance, a “m5.12xlarge” instance with 48 “vCPU”, 192 GB of memory, and enhanced network connection capacity would be sufficient to provide simultaneous computing capability for at least 48 workers. Hence, the scanning process could be completed by employing only 21 “m5.12xlarge” instances for less than one day, at a cost of approximately one thousand dollars (assuming $2.304 per instance per hour according to Amazon on-demand instance price [42]). Such a large-scale attack can also be deployed over PlanetLab [43] for free.

Furthermore, although the brute-force scanning process requires no prior knowledge of the real MAC address distribution among the entire search space, a screening process could, in practice, be accelerated by utilizing MAC address distribution patterns. One may conduct a sampling screening process in a selected reduced search space, with the purpose of estimating real MAC address distributions. If any distribution pattern other than a uniform one is identified, particularly those with apparent clustering features, it is possible to apply a heuristic search algorithm to accelerate the scanning process, providing that the goal of scanning is to detect a sufficiently large number of new valid sensor MAC addresses rather than a complete list of all sensor MAC addresses. In this manner, the overall scanning time could be further decreased.

## 4. Discussion

In this section, we discuss how to defend against the three attacks introduced in Section 2.4.

We first look at the defense to the MAC address enumeration attack. Recall when a sensor sends data to the server, the message contains the MAC address for sensor identification. However, since the MAC address is predictable, it is inappropriate for fulfilling such a purpose. Instead, a long random number can be used to identify the sensor, denoted as device ID. Even if the adversary knows one device ID, he/she cannot predict other device IDs given that the space of device IDs is too huge for enumeration.

We now look at the defense to the wardriving attack. If a random device ID is used, the wardriving attack becomes ineffective if the network is encrypted. For example, WPA2 should be used to protect WiFi, and HTTPS should be used for end-to-end encryption. Therefore, the adversary cannot obtain the decrypted content of the communication for the device ID.

A malicious user possessing an a.com sensor is dangerous. He/she may register the sensor following a.com’s procedure with a fake geographical location. The malicious user can also perform the MITM attack to manipulate the sensor data. Registration with a fake location could be avoided by an internal GPS module or via WiFi localization. To defeat the MITM attack, the certificate based mutual authentication can be used. That is, the sensor authenticates the servers and the servers authenticate the sensor as well. A private key has to be securely stored in the sensor. The malicious user should not be able to change the firmware of the sensor either.

In summary, we believe that a sensor should adopt the following strategies for sensor data security and integrity. Secure boot should be used to prevent the manipulation of the firmware of the sensor. With secure boot, if the firmware is changed, the sensor will not boot. Such a firmware is trustworthy. Flash encryption should be used to protect sensitive data on the flash, including the WiFi credentials. Certificate based mutual authentication with TLS should be used to defeat the MITM attack and protect the communication. With mutual authentication, the server can identify the sensor and a random device ID may not be necessary since the server only accepts data from authenticated sensors. The mutual authentication renders the MITM attack invalid and the hash of the sensor’s public key can be adopted as the device ID if needed. Secure storage should be used to store the sensor’s private key so that the adversary cannot obtain the private key for the MITM attack. Sensors should have different private keys so that even if one sensor is compromised, it will not affect others. The location of the sensor can be obtained from either a GPS module on the sensor or WiFi localization. A GPS module can be problematic since a dedicated adversary may replace the module with an artificial one. The GPS may not work inside buildings. The WiFi localization may be more appropriate since the trusted firmware will retrieve the WiFi information for the purpose of localization. The server may also validate the reported location from the device via the IP location service [44], which finds the geolocation of a sensor from the IP address of the sensor while the accuracy of the IP location service is limited [45]. Secure firmware upgrade is needed in case that vulnerabilities are found in the system. The advance of hardware now actually makes the defense strategies introduced above possible for sensors using low-cost microcontrollers (MCUs) [46,47,48,49].

## 5. Conclusions

In this paper, we perform a systematic analysis of security and data integrity of a popular air quality monitoring system, a.com, which uses low-cost air quality sensors to gather and remotely manage pollutant concentration data. By analyzing the traffic of sensors, we are able to understand the architecture of the a.com system and its communication protocol. We then present approaches of polluting sensor data in three scenarios: sensor in physical possession, knowing sensor MAC addresses (or geographical location), and automatic large-scale system pollution. By designing several attack methods including man-in-the-middle attack, spoofing attack, wardriving attack, and device scanning attack, we were able to successfully pollute sensor data with fabricated data. We also demonstrated that we have the capability of polluting data from any sensors of interest that are deployed globally without being detected by the owners of sensors. Guidelines on security improvement and defense mechanisms to mitigate such system vulnerabilities are also provided and discussed.

Low-cost and connected air quality monitoring networks have gained tremendous amount of popularity recently due to their transformative role in air quality management. However, security is rarely considered in the design and deployment of low-cost air quality sensor networks. Our research results demonstrate the potentially catastrophic consequences of neglecting security features on such sensor networks, and highlight the urgent need to enhance cyber security on operational air quality monitoring networks using connected low-cost sensors. To the best of our knowledge, this is the first systematic analysis in the field of air quality sensor network security. Findings from this study have important implications for designing the next generation air quality sensor network. We also provide guidelines of designing a secure air quality monitoring system.

## Figures and Tables

**Table 1:** Data format description for M-1A and M-1B.

HTTP Request Header	Description	HTTP Request Header	Description
mac	MAC address	pm2_5_atm	PM2.5 w/ correction
lat	latitude of the sensor location	pm10_0_atm	PM10.0 w/ correction
lon	longitude of the sensor location	pm1_0_cf_1	PM1.0 w/o correction
key1	key K-1A (K-1B)	pm2_5_cf_1	PM2.5 w/o correction
key2	key K-2A (K-2B)	pm10_0_cf_1	PM10.0 w/o correction
uptime	uptime in sec	p_0_3_um	particles ≤ 0.3 μm count/dl
rssi	signal strength	p_0_5_um	particles ≤ 0.5 μm count/dl
current_temp_f	temperature	p_1_0_um	particles ≤ 1.0 μm count/dl
current_humidity	humidity	p_2_5_um	particles ≤ 2.5 μm count/dl
current_dewpoint_f	dewpoint temperature	p_5_0_um	particles ≤ 5.0 μm count/dl
pressure	pressure	p_10_0_um	particles ≤ 10.0 μm count/dl
pm1_0_atm	PM1.0 w/ correction		

**Table 2:** Data format description for M-2A and M-2B.

HTTP Request Header	Description
key	ThingSpeak key K-1A (K-1B) for identification
field1 - field8	a part of sensor measurements

**Table 3:** Data format description for M-3A and M-3B.

HTTP Request Header	Description
key	ThingSpeak key K-2A (K-2B) for identification
field1 - field10	the other part of sensor measurements

**Table 4:** Response format from the www.a.com server.

Scenarios	Response
MAC address and keys correct	geographic coordinates
MAC address correct but keys wrong	geographic coordinates and correct keys
MAC address wrong	NOT FOUND

**Table 5:** Discovered MAC address prefixes assigned to Espressif Systems.

MAC Prefixes	MAC Prefixes
18:FE:34	24:0A:C4
24:B2:DE	2C:3A:E8
30:AE:A4	3C:71:BF
54:5A:A6	5C:CF:7F
60:01:94	68:C6:3A
84:0D:8E	84:F3:EB
90:97:D5	A0:20:A6
A4:7B:9D	AC:D0:74
B4:E6:2D	BC:DD:C2
C4:4F:33	CC:50:E3
D8:A0:1D	DC:4F:22
EC:FA:BC	80:7D:3A

**Table 6:** Theoretical efficiency of MAC address scanning with different number of parallel computing workers.

**NO. of Workers**	5	100	300	500	800	1000
**MAC Scanned Per Hour**	$9\times 10^{4}$	$1.8\times 10^{6}$	$5.4\times 10^{6}$	$9\times 10^{6}$	$1.44\times 10^{7}$	$1.8\times 10^{7}$
**Approximated Total Hours**	4474	224	75	45	28	23

## Nomenclature

**Air Quality Index**	
AQI	Air quality index
*C*	Pollutant concentration
$C_{low}$	Concentration breakpoint that is ≤C
$C_{high}$	Concentration breakpoint that is ≥C
$I_{low}$	Index breakpoint corresponding to $C_{low}$
$I_{high}$	Index breakpoint corresponding to $C_{high}$
	
**Data Pollution**	
$I_{VU}$	Update interval of the visualized data on map
$I_{r}$	Real sensor data update interval
$I_{f}$	Fake sensor data update interval
$P_{r}$	Real PM2.5 measurement
$P_{f}$	Fabricated PM2.5 measurement
$R_{\mathrm{PM}2.5}$	Corresponding range of PM2.5 with respect to the targeted AQI
LB	Lower bound of $R_{\mathrm{PM}2.5}$
UB	Upper bound of $R_{\mathrm{PM}2.5}$
$I_{max}$	The maximum value of $I_{f}$
$I_{uni}$	Uniform fake sensor data update interval
*n*	Assumed number of sensors being polluted simultaneously
$P_{r_{1}}$ − $P_{r_{n}}$	Corresponding real PM2.5 measurements for sensors being polluted simultaneously
$N_{1}$	Number of sensors that have real PM2.5 measurements less than LB
$N_{2}$	Number of sensors that have real PM2.5 measurements less than UB
$S_{1}$	Set $\left\{1,\cdots ,N_{1}\right\}$
$S_{2}$	Set $\left\{N_{2}+1,\cdots ,n\right\}$
$I_{uni-S_{1}}$	Uniform fake sensor data update interval of set $S_{1}$
$I_{uni-S_{2}}$	Uniform fake sensor data update interval of set $S_{2}$
	
**Message Composition**	
M−1A	The first message sent from the sensor winthin one period
M−2A	The second message sent from the sensor winthin one period
M−3A	The third message sent from the sensor winthin one period
M−1B	The fourth message sent from the sensor winthin one period
M−2B	The fifth message sent from the sensor winthin one period
M−3B	The sixth message sent from the sensor winthin one period
K−1A	The first ThingSpeak key for channel A
K−2A	The second ThingSpeak key for channel A
K−1B	The first ThingSpeak key for channel B
K−2B	The second ThingSpeak key for channel B

## References

[1] Burnett R., Chen H., Szyszkowicz M., Fann N., Hubbell B., Pope C.A., Apte J.S., Brauer M., Cohen A., Weichenthal S. (2018). Global estimates of mortality associated with long-term exposure to outdoor fine particulate matter. Proc. Natl. Acad. Sci. USA.

[2] Lim S., Vos T., Flaxman A., Danaei G., Shibuya K., Adair-Rohani H., Amann M., Anderson H., Andrews K., Aryee M. (2012). A comparative risk assessment of burden of disease and injury attributable to 67 risk factors and risk factor clusters in 21 regions, 1990–2010: A systematic analysis for the Global Burden of Disease Study 2010. Lancet.

[3] Wei W., Ramalho O., Mandin C. (2015). Indoor air quality requirements in green building certifications. Build. Environ..

[4] Fuzzi S., Baltensperger U., Carslaw K., Decesari S., Denier Van Der Gon H., Facchini M., Fowler D., Koren I., Langford B., Lohmann U. (2015). Particulate matter, air quality and climate: Lessons learned and future needs. Atmos. Chem. Phys..

[5] Jones A.P. (1999). Indoor air quality and health. Atmos. Environ..

[6] Neira M., Prüss-Ustün A., Mudu P. (2018). Reduce air pollution to beat NCDs: From recognition to action. Lancet.

[7] Hall E.S., Kaushik S.M., Vanderpool R.W., Duvall R.M., Beaver M.R., Long R.W., Solomon P.A. (2014). Integrating sensor monitoring technology into the current air pollution regulatory support paradigm: Practical considerations. Am. J. Environ. Eng..

[8] Apte J.S., Messier K.P., Gani S., Brauer M., Kirchstetter T.W., Lunden M.M., Marshall J.D., Portier C.J., Vermeulen R.C., Hamburg S.P. (2017). High-resolution air pollution mapping with Google street view cars: Exploiting big data. Environ. Sci. Technol..

[9] Karner A.A., Eisinger D.S., Niemeier D.A. (2010). Near-roadway air quality: Synthesizing the findings from real-world data. Environ. Sci. Technol..

[10] Jiao W., Hagler G., Williams R., Sharpe R., Brown R., Garver D., Judge R., Caudill M., Rickard J., Davis M. (2016). Community Air Sensor Network (CAIRSENSE) project: Evaluation of low-cost sensor performance in a suburban environment in the southeastern United States. Atmos. Meas. Tech..

[11] Snyder E.G., Watkins T.H., Solomon P.A., Thoma E.D., Williams R.W., Hagler G.S.W., Shelow D., Hindin D.A., Kilaru V.J., Preuss P.W. (2013). The Changing Paradigm of Air Pollution Monitoring. Environ. Sci. Technol..

[12] Michel G., Laurent S., Annette B. (2017). Measuring Air Pollution with Low-Cost Sensors.

[13] Spinelle L., Gerboles M., Villani M.G., Aleixandre M., Bonavitacola F. (2015). Field calibration of a cluster of low-cost available sensors for air quality monitoring. Part A: Ozone and nitrogen dioxide. Sens. Actuators B Chem..

[14] Spinelle L., Gerboles M., Villani M.G., Aleixandre M., Bonavitacola F. (2017). Field calibration of a cluster of low-cost commercially available sensors for air quality monitoring. Part B: NO, CO and CO_2_. Sens. Actuators B Chem..

[15] Spinelle L., Gerboles M., Kok G., Persijn S., Sauerwald T. (2017). Review of portable and low-cost sensors for the ambient air monitoring of benzene and other volatile organic compounds. Sensors.

[16] Cavaliere A., Carotenuto F., Di Gennaro F., Gioli B., Gualtieri G., Martelli F., Matese A., Toscano P., Vagnoli C., Zaldei A. (2018). Development of Low-Cost Air Quality Stations for Next Generation Monitoring Networks: Calibration and Validation of PM2.5 and PM10 Sensors. Sensors.

[17] Gubbi J., Buyya R., Marusic S., Palaniswami M. (2013). Internet of Things (IoT): A vision, architectural elements, and future directions. Future Gener. Comput. Syst..

[18] Atzori L., Iera A., Morabito G. (2010). The internet of things: A survey. Comput. Netw..

[19] Yi W.Y., Lo K.M., Mak T., Leung K.S., Leung Y., Meng M.L. (2015). A survey of wireless sensor network based air pollution monitoring systems. Sensors.

[20] English P.B., Olmedo L., Bejarano E., Lugo H., Murillo E., Seto E., Wong M., King G., Wilkie A., Meltzer D. (2017). The Imperial County Community Air Monitoring Network: A model for community-based environmental monitoring for public health action. Environ. Health Perspect..

[21] Amegah A.K. (2018). Proliferation of low-cost sensors. What prospects for air pollution epidemiologic research in Sub-Saharan Africa?. Environ. Pollut..

[22] Chahid Y., Benabdellah M., Azizi A. Internet of things security. Proceedings of the 2017 International Conference on Wireless Technologies, Embedded and Intelligent Systems (WITS).

[23] Kumar J.S., Patel D.R. (2014). A survey on internet of things: Security and privacy issues. Int. J. Comput. Appl..

[24] Suo H., Wan J., Zou C., Liu J. Security in the internet of things: A review. Proceedings of the 2012 International Conference on Computer Science and Electronics Engineering (ICCSEE).

[25] Sicari S., Rizzardi A., Grieco L.A., Coen-Porisini A. (2015). Security, privacy and trust in Internet of Things: The road ahead. Comput. Netw..

[26] Miorandi D., Sicari S., De Pellegrini F., Chlamtac I. (2012). Internet of things: Vision, applications and research challenges. Ad Hoc Netw..

[27] Medaglia C.M., Serbanati A. (2010). An overview of privacy and security issues in the internet of things. The Internet of Things.

[28] Ling Z., Luo J., Xu Y., Gao C., Wu K., Fu X. (2017). Security Vulnerabilities of Internet of Things: A Case Study of the Smart Plug System. IEEE Internet Things J. (Iot-J).

[29] Ling Z., Liu K., Xu Y., Jin Y., Fu X. An End-to-End View of IoT Security and Privacy. Proceedings of the 60th IEEE Global Communications Conference (Globecom).

[30] Srivatsa P., Pandhare A. (2016). Indoor Air Quality: IoT Solution. Int. J. Res. Advent Technol..

[31] Marques G., Pitarma R. (2016). An indoor monitoring system for ambient assisted living based on internet of things architecture. Int. J. Environ. Res. Public Health.

[32] Salamone F., Belussi L., Danza L., Galanos T., Ghellere M., Meroni I. (2017). Design and development of a nearable wireless system to control indoor air quality and indoor lighting quality. Sensors.

[33] Bhattacharya S., Sridevi S., Pitchiah R. Indoor air quality monitoring using wireless sensor network. Proceedings of the 2012 Sixth International Conference on Sensing Technology (ICST).

[34] Pope C.A., Dockery D.W. (2006). Health effects of fine particulate air pollution: Lines that connect. J. Air Waste Manag. Assoc..

[35] Cortesi A., Hils M., Kriechbaumer T. (2010). mitmproxy: A Free and Open Source Interactive HTTPS Proxy. https://mitmproxy.org/.

[36] Song D. Dsniff. https://www.monkey.org/~dugsong/dsniff/.

[37] Wireshark. https://www.wireshark.org/.

[38] Air Quality Index—Wikipedia. https://en.wikipedia.org/wiki/Air_quality_index.

[39] Kismet: A Wireless Network Detector, Sniffer, and Intrusion Detection System. https://www.kismetwireless.net/.

[40] Wireshark OUI Lookup Tool. https://www.wireshark.org/tools/oui-lookup.html.

[41] Amazon EC2. https://aws.amazon.com/ec2/.

[42] Amazon EC2 Pricing. https://aws.amazon.com/ec2/pricing/on-demand/.

[43] PlanetLab: An Open Platform for Developing, Deploying, and Accessing Planetary-Scale Services. https://www.planet-lab.org/.

[44] Brand Media, Inc. Where is Geolocation of an IP Address?. https://www.iplocation.net/.

[45] Center for Applied Internet Data Analysis Internet Protocol Address (IP) Geolocation Bibliography. http://www.caida.org/projects/cybersecurity/geolocation/bib/.

[46] The Two-Dollar Secure IoT Solution: Mongoose OS + ESP8266 + ATECC508 + AWS IoT. https://mongoose-os.com/blog/mongoose-esp8266-atecc508-aws/.

[47] Espressif Systems (Shanghai) PTE LTD ESP-IDF Programming Guide. https://docs.espressif.com/projects/esp-idf/en/latest/.

[48] Microchip Technology Inc. SAML11 Xplained Pro Evaluation Kit. http://www.microchip.com/DevelopmentTools/ProductDetails/dm320205.

[49] Texas Instruments Incorporated CC3220 SimpleLink Wi-Fi and IoT, Single-Chip Wireless MCU Solution. http://www.ti.com/product/CC3220?keyMatch=cc3220sf&tisearch=Search-EN-Everything.

